# What's New in Endocrinology: The Chromaffin Cell

**DOI:** 10.3389/fendo.2018.00711

**Published:** 2018-12-04

**Authors:** Lee E. Eiden, Sunny Zhihong Jiang

**Affiliations:** Section on Molecular Neuroscience, Laboratory of Cellular and Molecular Regulation, Intramural Research Program, National Institute of Mental Health, Bethesda, MD, United States

**Keywords:** chromaffin cell, catestatin, PACAP, interleukin 6, gap junction, NCS-Rapgef2

## Abstract

Recent advances in understanding the intracellular and intercellular features of adrenal chromatin cells as stress transducers are reviewed here, along with their implications for endocrine function in other tissues and organs participating in endocrine regulation in the mammalian organism.

## Introduction

It is sometimes useful to view the sprawling field of endocrinology through a narrow window to view advances that might otherwise be lost in the larger and more chaotic picture. Here, we take the opportunity to review briefly recent advances in our understanding of the regulation of the mammalian adrenal medulla, the storehouse for secretion of epinephrine that is critical for cardiovascular, neuronal, and metabolic homeostatic control, especially during stress. We compare the accepted view of the adrenal medulla of the twentieth century, still promulgated in most textbooks, and what we know about its function given recent information over the past decade or so. In each of five areas, we present “foundational” data, and then review emerging information sets that have significantly changed how the adrenal medulla is viewed as a stress-transducing endocrine tissue, and in addition shed significant light on the function of other mammalian endocrine organs and systems.

**Figure 1:**
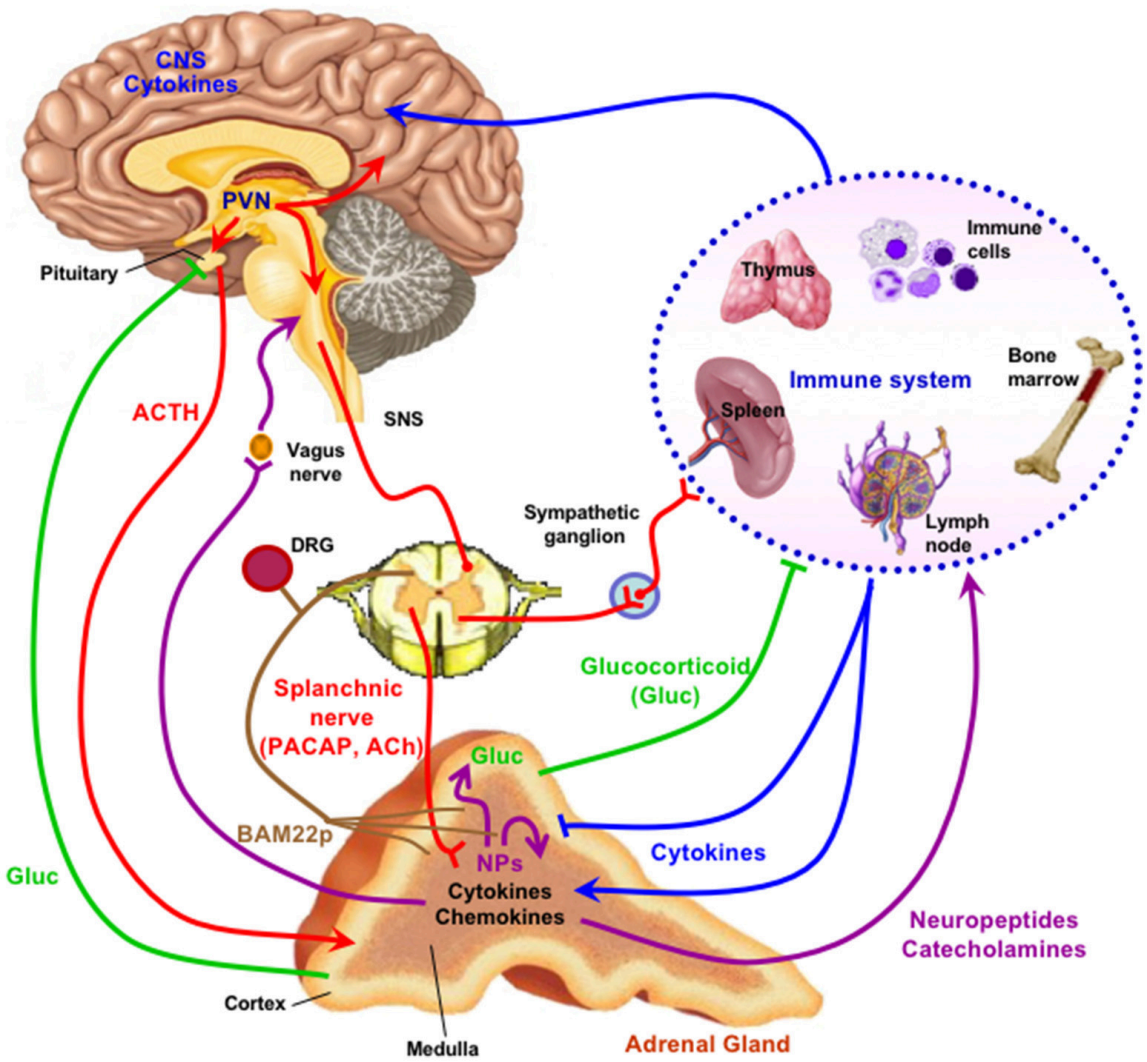
The adrenal medulla as a stress transducer and neuroimmunoinflammatory and cardiovascular regulator. Cytokines are delivered to receptors on chromaffin cells as blood-borne messengers or via mobile secreting cells such as monocytes/macrophages; neurotransmitters PACAP and ACh are delivered to receptors on chromaffin cells via the splanchnic nerve. Activation of chromaffin cell signaling pathways (see Figure 2) increase expression of genes encoding neuropeptides (NPs), other mediators, catecholamine biosynthetic enzymes, and adhesion factors and connexins that increase cell-cell communication among chromaffin cells, and amplify catecholamine, neuropeptide, and chromogranin output in response to stress. Secreted neuropeptides such as galanin may in turn act upon the adrenal cortex to modulate glucocorticoid output; catecholamines act as hormones at cardiovascular and other targets; chromogranins and their derived peptides have both autocrine and paracrine actions in the adrenal medulla and also as hormones; BAM-22P may act on receptors on sensory neurons innervating the adrenal gland. For further explication of the figure, see the text. Note that there is some degree of species specificity to expression of cytokines and neuropeptides in adrenal medulla, so that the schematic offered here may differ in some particulars depending on the species, including *H. sapiens*, under consideration (1–8). Figure adapted from (9).

The first area in which significantly new developments in our understanding of chromaffin cell function have occurred is the emergence of the neuropeptide PACAP, rather than acetylcholine, as the physiologically dominant splanchnicoadrenomedullary synaptic neurotransmitter during stress. A second is the discovery in adrenal medulla of a neuroendocrine-specific cyclic AMP effector that propagates signaling for gene regulation in response to stress into the chromaffin cell. Third, peptides found in adrenal medulla and released from it may have important paracrine and autocrine roles, while their roles as both biomarkers and physiological actors is still unfolding. A fourth emerging concept is that chromaffin cells of the adrenal medulla work together via altered gap junction coupling and cellular adhesion during certain physiological states, predominantly in stress. Finally, we are becoming aware, with acquisition of new *in vivo* and cell culture data, that the adrenal medulla is not only the original “stress transducer” of the body, but also a regulatory nexus for the secretion of peptides important in stress-inflammation interactions and in integration of stress responding with the sensory nervous system. We will take each of these points in order, and end by summarizing how an updated view of adrenomedullary function has illuminated endocrine cell and organ function in general (Figure 1).

## Pacap as a Stress Transmitter at the Splanchnicoadrenomedullary Synapse

Almost 70 years ago, Rex Coupland properly defined the first peripheral synapse, through electron microscopical observation, detailing the innervation of chromaffin cells of the golden hamster adrenal medulla by the cholinergic sympathetic pre-ganglionic fibers of the splanchnic nerve (10). Somewhat earlier, the storage of epinephrine (and norepinephrine) within secretory granules was characterized (11). The morphological, cell biological, and biochemical aspects of chromaffin cell function were integrated by several additional observations. These included the ultrastructural observation of the epinephrine-containing secretory granules presenting an omega-shaped profile to the extracellular space of the chromaffin cell by Coupland; the determination that a large protein (chromogranin A) was contained in and secreted from the adrenal medulla upon splanchnic nerve stimulation [see for original pertinent literature (12, 13)]^1^; and finally the observation that cholinergic and nerve stimulation was calcium-dependent (14). A rich cascade of concepts followed, and culminated in Douglas's idea of stimulus-secretion coupling (15), in analogy to muscle stimulus-contraction coupling. In this case, a transmitter released from the splanchnic nerve excited the chromaffin cell through an ionotropic receptor, calcium influx occurred, and exocytosis followed, with the release of granular proteins and catecholamines into the circulation. The transmitter released from the splanchnic nerve (and at all other preganglionic sympathetic/sympathoadrenal synapses) of course was acetylcholine. It caused cellular depolarization through the nicotinic cholinergic receptor, and exocytotic secretion ensued (16). Subsequently, the splanchnicoadrenomedullary synapse became the paradigm for peripheral neurotransmission at sympathetic as well as parasympathetic ganglia.

The ability of acetylcholine to generate an action potential by acting upon sodium channels on chromaffin cells (and post-ganglionic sympathetic and parasympathetic neurons) explained its secretagogue activity for epinephrine release from the adrenal medulla (16). Similar nicotinic receptor activation on post-ganglionic sympathetic and parasympathetic neurons was responsible for norepinephrine and acetylcholine release (respectively) at autonomic neuroeffector junctions in heart, spleen, lymphatic tissue, and elsewhere throughout the body. The possibility for additional complexity, however, in the neurotransmitter coding of the autonomic nervous system began to arise in the late 70s with several laboratories exploring the long-term changes in chromaffin cells driven by stress-induced catecholamine secretion. Wakade and colleagues discovered that the adrenal medulla may release its entire complement of catecholamines during a bout of secretion lasting several hours, yet remain competent for secretion afterwards due to heroic repletion of the gland: at the end of a secretory bout that releases all of the epinephrine present in the adrenal medulla at the start of secretion, there remains as much epinephrine as before (17). The gland re-synthesizes its entire store of accumulated epinephrine in a few hours. How does this occur? Costa and colleagues explored the induction of the rate-limiting enzyme for catecholamine biosynthesis, tyrosine hydroxylase (TH), and determined that through both transcriptional and post-translation activation mechanisms, long-term splanchnicoadrenomedullary stimulation would induce TH and this would in turn increase the rate of catecholamine biosynthesis in a compensatory way (18). Possible mechanisms for this (since nicotinic stimulation was limited in its ability to induce TH as well as peptide synthesis in the gland) included the activation of muscarinic cholinergic receptors (19). Thoenen et al., however, noted that reflex stimulation of chromaffin cells *in situ* by whole-animal treatment with reserpine caused an induction of TH that could not be blocked with nicotinic or muscarinic blockers, and opined that either a novel type of cholinergic receptor must exist or, presciently, that a non-cholinergic substance might be released along with acetylcholine to allow TH induction (20). Ip and Zigmond posited something similar at sympathetic ganglion, where stimulation of the sympathetic trunk at 10 Hz (frequency typical of the stress response) caused in the superior cervical ganglion the induction of TH by a mechanism that could be pharmacologically explained only partially through an action of acetylcholine release from preganglionic terminals (21). The situation languished until the discovery of the neuropeptide pituitary adenylate cyclase-activating polypeptide (PACAP) (22) by Miyata et al. Despite its name, and its discovery as a hypothalamic peptide able to stimulate cAMP production in perfused anterior pituitary hemi-organs (23), three remarkable properties of PACAP were soon discovered. First, it was ubiquitously expressed in brain, and not only hypothalamus; second, it was present in peripheral tissues; third, application of PACAP in various contexts potently stimulated epinephrine secretion from the adrenal gland *in vivo*, from perfused adrenal gland *ex vivo*, and from chromaffin cells in culture [(24–27) and references therein].

The development of PACAP-deficient mice allowed characterization of antibodies that were unambiguous in detecting PACAP (compared to its structurally similar congener VIP) in the cholinergic nerve terminals of the splanchnic nerve within the adrenal gland (28). The PACAP knock-out mouse also allowed examination of the role of PACAP on adrenomedullary performance *in vivo*. Reflex stimulation of the splanchnic nerve after hypoglycemia induced by insulin shock increased plasma epinephrine levels, and adrenomedullary TH activity: both effects were blunted or abolished in PACAP-deficient mice, and correction of hypoglycemia did not occur, a fatal outcome unless prevented by intraperitoneal injection of PACAP or glucose itself (28). Later experiments conducted in adrenal slices *ex vivo* showed that epinephrine secretion upon splanchnic nerve stimulation at high frequency, but not at low stimulation rates, does not occur in slices from PACAP-deficient mice or from wild-type mice when perfused with the PACAP antagonist PACAP(6-38) (29, 30).

**Figure 2:**
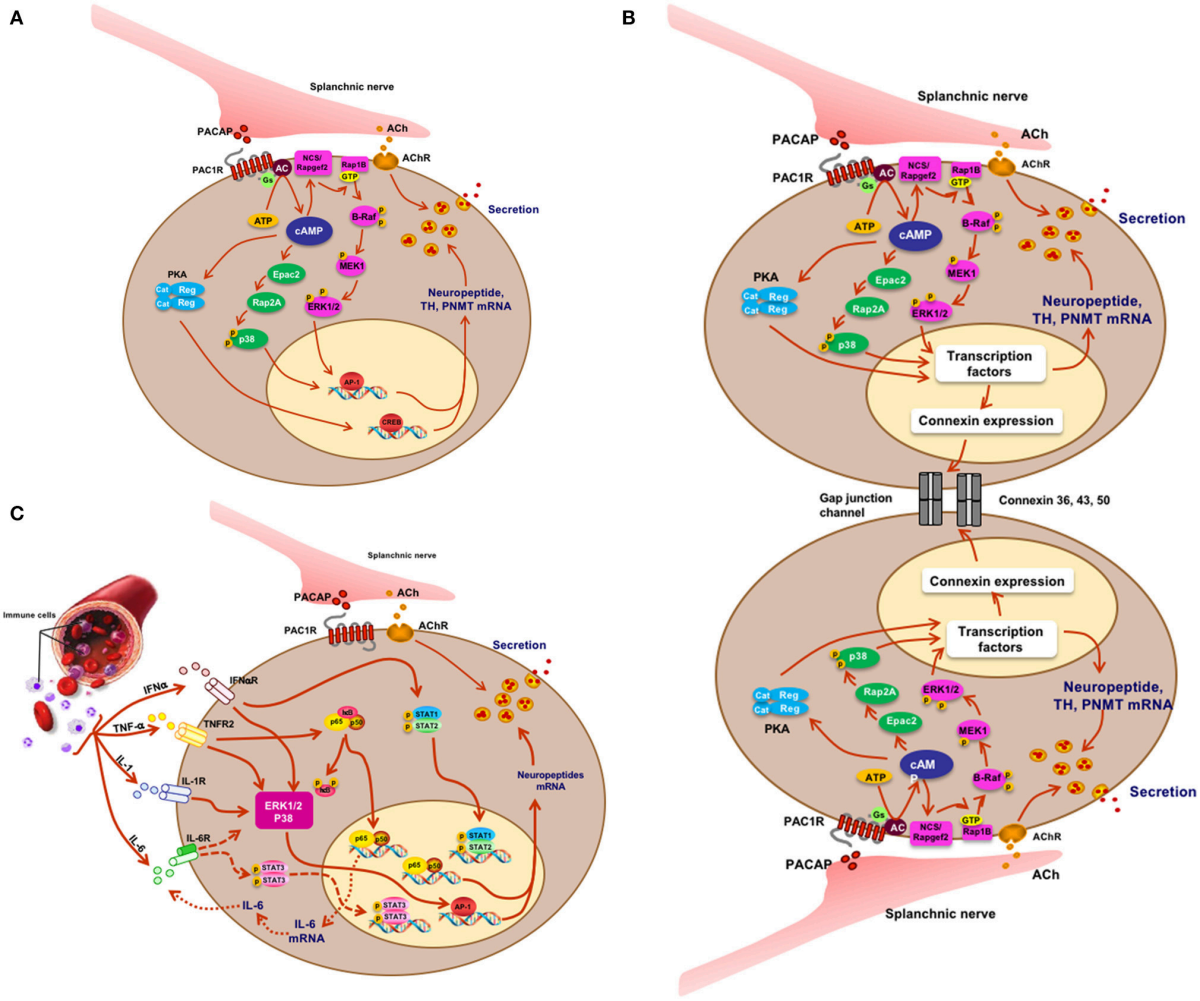
Signaling and physiology of the chromaffin cell. **(A)** Acetylcholine and PACAP (sympathetic pre-ganglionic) release from splanchnic nerve and stimulus-secretion-synthesis coupling in the chromaffin cell. **(B)** Mechanistic depiction of enhanced cell-cell interaction and hormone secretion under conditions of stress in the chromaffin cell. There is as yet no direct proof for PACAP or acetylcholine as the principal mediator of what might be termed the “gap junction response.” **(C)** IFN, TNF, IL-1, and IL-6 effects on cognate receptors on chromaffin cells and cellular sequelae in the chromaffin cell. Splanchnic nerve input affords synergistic as well as antagonistic interactions between cytokines and PACAP under physiological conditions in which both stress and inflammation may play a role. Figure adapted from (9).

What have we learned? First, PACAP is present at each of the splanchnic nerve terminals that contain cholinergic secretory vesicles and innervate the mouse adrenal chromaffin cells. Second, stress-induced catecholamine secretion and biosynthetic enzyme regulation is PACAP-dependent, regardless of whether the stressor is metabolic/systemic (e.g., insulin-induced hypoglycemia) or exteroceptive/psychogenic (e.g., restraint stress). Second, this dependence can be shown to exist at the level of the adrenal gland itself, since adrenal slices taken from PACAP-deficient mice, in which the morphology and neurochemistry of the splanchnicoadrenomedullary synapse is normal, fail to release epinephrine in response to high-frequency stimulation of the splanchnic nerve stump, while basal (low-frequency) catecholamine secretion is unaffected. These experiments appear to satisfy the criterion for PACAP as the neurotransmitter responsible for stress transduction at the adrenomedullary synapse (see Figure 2A) (31, 32).

How generalizable is the concept that PACAP, and not acetylcholine, is the major autonomic stress neurotransmitter? This involves answering two questions. First, in how many other mammalian species is PACAP the splanchnicoadrenomedullary transmitter? Second, is PACAP the stress transducing transmitter at other autonomic synapses of the sympathetic and parasympathetic nervous systems? With respect to the first question, catecholamine secretion is provoked by PACAP, either *in vivo* or in chromaffin cells in culture, in all mammals examined so far, including mice, rats, cows, dogs, and humans [reviewed in (27)]. Regarding the second, there is a significant body of evidence for receptivity of sympathetic post-ganglionic neurons, in culture, to PACAP, as evidenced by enhanced secretion as well as signaling for gene expression (33, 34). Post-ganglionic neurons of the system, notably in the heart, are also PACAP responsive (35). Thus, findings in the findings in the adrenal medulla likely herald a new perspective in adding the neuropeptide PACAP as the third major neurotransmitter of the autonomic nervous system, in addition to acetylcholine and norepinephrine.

## Gs-Gpcr Signaling for Secretion and Biosynthesis in the Chromaffin Cell

Hormone-secreting endocrine tissues must maintain their stores of secreted material, catecholamines from chromaffin cells, insulin from beta cells of the pancreas, hypophyseal hormones from their depots in anterior pituitary cells, incretins from endocrine cells of the gut, and so forth, in order to maintain patency for future endocrine response. This can occur via parallel but uncoupled processes if hormone secretion and biosynthesis both occur at about the same constitutive rate, but this is rarely the case, as hormone secretion tends to be episodic in response to metabolic and other homeostatic organismic demands, when the endocrine cell is called upon by first messenger secretagogues to release its characteristic hormone into the bloodstream to act at distant sites. Timely repletion of hormone content in endocrine tissue following a bout of secretion in these cases can occur in one of two ways. The first is a chronic overproduction of prohormone protein by the endocrine cell relative to storage capacity. In this case, excess hormone is degraded at times of low secretory demand, and diverted to the secretory pathway at times of high secretory demand. This type of regulation appears to be the case for chromogranin A in adrenal medulla: because chromogranin A is a constituent of secretory granule biogenesis itself, the chromaffin cell makes no more chromogranin than it does secretory granules. When the secretory granule complement is complete, excess chromogranin is apparently degraded in the trans-Golgi network and when the granule complement is depleted by a bout of secretion, this excess is re-directed toward granule production, so that chromogranin levels are maintained during secretion even when there is no compensatory up-regulation of chromogranin mRNA abundance compared to that of other co-secreted neuropeptides (36, 37). For (pro)hormones that are only a fraction of the total secretory granule protein content, however, a non-default regulatory mechanism for compensating hormone loss through secretion with enhanced biosynthesis must exist (this actually includes chromogranins themselves, in cells in which they are not the dominant secreted protein).

The concept of stimulus-secretion-synthesis coupling was introduced into the neuroendocrine lexicon with the realization that first messengers that act as secretagogues, facilitating the release of stored hormones into the bloodstream, may simultaneously signal to the endocrine cell to engage the genetic machinery that allows repletion of hormone (38). For hormones that are produced by prohormone processing within the secretory granule, this is reflected in enhanced transcription of the prohormone gene and subsequently enhanced translation of its mRNA into prohormone protein. In the adrenal medulla, in which secreted material is a mixture of protein (chromogranins and neuropeptides) and small molecules (catecholamines and ATP), stimulus-secretion synthesis coupling must accommodate both types of secreted material. Thus, secretagogue (first-messenger) signaling leads immediately to calcium influx, then to activation of the secretory apparatus and release from the cell of the contents of the secretory vesicle/granule. Simultaneously, downstream signaling from the first messenger must engage pathways leading to (i) stimulation of transcription of prohormone protein-encoding genes and (ii) stimulation of biosynthetic enzymes responsible for catecholamine and other small-molecule biosynthesis. In the case of catecholamine production, this means activation of both the enzymatic activity, via phosphorylation, of the rate-limiting enzyme for catecholamine biosynthesis, tyrosine hydroxylase (TH) and increased production of the TH protein itself, via increased transcription from the TH gene (39).

The detailed cellular mechanisms of signaling for hormone repletion in the adrenal medulla was much-studied throughout the Twentieth century and in many ways was paradigmatic for understanding this process in other endocrine cells and tissues. The second messenger most often evoked as mediating stimulus-synthesis coupling is calcium, the same second messenger that triggers secretin in most endocrine cells. However, in the adrenal medulla, cyclic AMP was implicated early on in both transcriptional and post-translation effects on TH activation. Later, examination of stimulus-secretion-synthesis coupling related to co-stored peptides such as substance P, NPY, enkephalin, and galanin revealed that while cyclic AMP appears (as in anterior pituitary cells such as corticotropes) to be the main second messenger for activation of gene transcription of the latter, the classical pathway to cAMP-dependent gene activation by the third messenger protein kinase A (PKA) was insufficient to explain how this signaling could occur.

The concept that cAMP's actions are mediated solely through activation of the serine/threonine protein kinase protein kinase A (PKA) was a durable one for several decades after its promulgation by Kuo and Greengard (40). However, toward the end of the Twentieth century it became increasingly apparent that not only could cAMP gate calcium channels on the surface of specialized cells, such as those of the olfactory mucosa (41), but that there were a plethora of cAMP-dependent and PKA-independent actions of first messengers that strongly suggested the existence of other cAMP effectors within mammalian, especially neuroendocrine cells. In the late 90s, two groups discovered two proteins among a family of Rapgef proteins (for guanine nucleotide exchange factors activating the signaling kinase Rap), variously called Epac1 and 2 (the currently most common name), or Rapgef3 and 4, or cAMP-GEF1 and −2 (42–44). These proteins clearly expanded the range of cAMP effectors within mammalian cells from protein kinases to guanine nucleotide exchange factors, with their own sets of downstream effectors (mainly MAP kinases) distinct from cytoplasmic substrates such as glycogen synthase, and nuclear transcription factors such as CREB, activated by PKA.

Our own laboratory noted in 2012 that galanin biosynthesis in the chromaffin cell was regulated by the stress-associated secretagogue PACAP, via a cyclic AMP signaling pathway independent of PKA, and requiring activation of the MAP kinase ERK (45). The cAMP effector linking activation of adenylate cyclase and ERK by PACAP was identified by both loss- and gain-of-function experiments, in cellula, as the gene product of the guanine nucleotide exchange factor Rapgef2 (46). This enzyme had been deemed to be insensitive to regulation by cAMP by both *in vitro* biochemical criteria and by sequence comparison to known cAMP binding proteins (47, 48). However, others had identified Rapgef2 as a cyclic nucleotide-regulated protein in neuroendocrine (melanoma) cells (49, 50). We have subsequently identified two mRNA variants transcribed from the Rapgef2 gene, which we have termed NN (for non-neuronal)-Rapgef2 and NCS (for neuritogenic cAMP sensor)-Rapgef2. The latter transcript appears to be generated exclusively in neuronal and endocrine cell types/tissues in adult rodents, and is responsible for linking cAMP elevation and ERK activation not only in chromaffin cells, but in neurons of the central nervous system (51). Re-assessment of cAMP signaling with respect to its parcellation between protein kinase A, and the guanine nucleotide exchange factors Epac and NCS-Rapgef2, is likely to occur throughout the field of endocrinology in the coming years. The characterization of the cellular assignments of these three cAMP effectors in signaling not only within the adrenal medulla, but in anterior pituitary, pancreatic islets, enterochromaffin cells, and other endocrine and neuronal cells is presently underway in several laboratories (51–56).

## The Role of Peptides Secreted From the Adrenal Medulla

As mentioned previously, the finding that the adrenal medulla secretes bioactive neuropeptides in addition to catecholamines was a major development for neurochemistry and neuroendocrinology in the early 1980s, and in fact paved the way for the discovery of the mRNAs encoding the opiate peptides (57–60). Its historical roots exist in the discovery of the chromogranins as secreted proteins of the adrenal medulla (vida infra); the discovery that enkephalin peptides are a major constituent of the secretory granules of the adrenal medulla (57, 58); and the realization that chromogranin A is itself a prohormone for bioactive peptides including pancreastatin, vasostatin, catestatin, and others (61–64). The adrenal medulla-specific proenkephalin-derived BAM-22P was identified via systematic peptidomics analysis of the adrenal medullary “peptide storehouse” (65). Adrenomedullin was discovered via a specific proteomics-based search for novel cAMP-elevating neuropeptides in pheochromocytoma peptide extracts (66). The presence of a rich secretory cocktail, comprising the chromogranin-derived, enkephalin-derived, and other neuropeptides, as well as the neuropeptides substance P, galanin, and NPY raised the important question of what the function of such released peptides might be. The answer(s) to these questions was initially frustrated by a lack of knowledge about where the corresponding receptors for peptides exist, and thus whether they were most likely to play an autocrine, paracrine, or hormonal function *in vivo* (63). The molecular nature of the receptor for catestatin, one of the CgA-derived peptides of the adrenal medulla, is still uncertain. However, O'Connor and colleagues were able to show convincingly that catestatin is able to modulate acetylcholine-induced catecholamine secretion from chromaffin cells, without affecting secretion caused by potassium or barium depolarization or ionophore-stimulated release, suggesting that its receptor either is, or acts immediately downstream of, the cholinergic nicotinic receptor of the chromaffin cell (67). More recently, it has been demonstrated in PC12 cells that catestatin affects not only acetylcholine-induced but also PACAP-induced catecholamine secretion, making its role in stress-related as well as basal adrenomedullary function of likely physiological importance (68). Likewise, substance P has been proposed as an autocrine regulator of catecholamine secretion and biosynthesis, based on its negative modulation of nicotinic receptor activation in chromaffin cells (69–71).

Catestatin, however, is truly protean in its autocrine and hormonal roles throughout the body. Perhaps the most striking finding about catestatin is its ability to rescue a chromogranin A-deficient phenotype in mice (72). Catestatin/chromogranin-mediated effects permeate all functions of the adrenal chromaffin cell (72). This includes the formation of secretory granules themselves (73), although this function appears to be shared with other granin proteins in chromaffin cells (74), and modulation of catecholamine release (75). Physiologically, catestatin extends the range of the adrenal medulla as an endocrine organ beyond the scope of catecholamine effects on cardiovascular function and metabolism, to endocrine (catestatin) modulation of the secretion, metabolism, and morphology of endocrine cells which are its targets (including the chromaffin cell) as well as its effects on modulation of beta cell function, immune regulation, and muscle and neuronal cellular metabolism (76, 77). As well, several other chromogranin A-derived peptides, including vasostatin, have potent cardiovascular effects that make the adrenal medulla a multi-pronged regulator of cardiovascular as well as metabolic function, and therefore link stress transduction even more tightly to cardiovascular and metabolic physiology (78).

The protean role of chromogranin-derived peptides, at every level of adrenomedullary function, has opened up an important chapter of endocrinology by sharpening appreciation for the role of peptide hormones, and their receptors, as highly adaptive components of the evolution of the endocrine network through constant adaptation of peptides to new regulatory roles, especially through tissue-specific expression of various peptides due to cell-specific prohormone processing. Such is the case of BAM22P. This 22-amino acid proenkephalin-derived peptide (YGGFMRRVGRPEWWMDYQKRYG) is found almost exclusively in the adrenal medulla, most likely because the only partial processing of proenkephalin in the adrenal gland, unlike in neuronal cells, results in a plethora of “incompletely” processed peptides. These however, are not only intermediates. Only distantly related to the mu opiate receptor, which is mainly liganded by leu- and met-enkephalin *in vivo*, the BAM22P receptor shows a more than 50-fold higher affinity for BAM22P than for met-enkephalin, which is contained within the primary sequence of the BAM22P peptide (79). The exclusive presence of the BAM22P receptor in sensory neurons, and the high levels of BAM22P in adrenal medulla, suggests that this peptide may be a specific first messenger for adrenomedullary communication with sensory nerves that abundantly innervate the adrenal gland (Figure 1). A link between the major stress-transducing endocrine organ, the adrenal medulla, and the sensory nervous system at this anatomical locus is intriguing to consider.

## The Concept of Organ Plasticity in Stress Responding of the Adrenal Medulla

It is by now well-known that endocrine tissues develop as “colonies” of cells devoted to a single secretory mission, but it was initially thought that each cell within this colony acts more-or-less independently of its neighbors in responding to first-messenger secretagogues with hormone release: a massively parallel rather than a highly integrated response. In the secretion of vasopressin, however, several investigators promulgated the concept of “concerted” actions of the entire secretory cell cohort (80). This seemed to account for the dynamics of secretion in this system more comprehensively, and mechanisms of this type are now being explored and discovered in other cell types. “Concerted secretion” could occur by one of two (or both) mechanisms. One of these is paracrine/autocrine regulation, in which substances secreted from a single secretory cell interact with receptors on the same cell (autocrine regulation) or neighboring cells of the same (also autocrine) or another (paracrine) type, to modulate their secretion. In any event, the concept of autocrine/paracrine regulation was easily applied to the adrenal medulla, in part to explain the actions of hormones besides catecholamines (see previous section) which appeared to be released from the adrenal medulla in too low levels to be bona fide hormones, thus begging for an additional function to explain their production in and secretion from chromaffin cells. A second mode of modulating the secretory response of endocrine cells as an organ collective consists in stimulus-dependent changes in cellular adhesion mediating cell-cell interactions called gap junctions that can affect secretory performance (Figure 2B). Gap junctions are electrical connections that lower the resistance between cells that are connected by them. Connexins are the proteins that make up the hexameric hemichannels that form gap junctions: when two hemichannels on adjacent cells are apposed, a gap junction is formed. In 2001 Martin et al. (81) made the seminar observation that connexins are expressed by rat adrenal chromaffin cells. The history of the gap junction in adrenal medulla and other endocrine tissues is capably summarized by Colomer et al. (82): suffice it to say here that the initial demonstration of the chromaffin cell gap channel was facilitated initially by the realization that the disparate cellular resistances of isolated chromaffin cells in culture are considerably greater than those of chromaffin cells in intact adrenomedullary cells, with these differences in input resistance logically inferred to arise from the existence of gap junctions between the latter and not the former (81, 83). A series of *in vivo* experiments have been performed by the Guerineau laboratory to assess the role(s) of gap junctions in adrenomedullary function *in vivo* [summarized in (84)]. Rat adrenomedullary slices were examined *ex vivo* both before and after 5 days of cold stress (85). Morphological remodeling of both splanchnicoadrenomedullary synapses and chromaffin cells at their borders of adjacency was observed, along with increased dye permeation between cells at gap junctions, and increased electrical coupling following exogenous depolarization of individual chromaffin cells. Further experiments have indicated that stress *in vivo* causes increased catecholamine secretion in response to electrical stimulation that is blocked by pharmacological inhibition of gap junction formation. Detail of biochemical constitution of gap junctions, and gap-junction dependence of stress-induced enhancement of catecholamine secretion from chromaffin cells *in vivo* have followed (82, 86). Clearly there is, in addition to a shift from predominantly cholinergic to PACAPergic neurotransmission at the adrenomedullary synapse from rest to stress upon initial exposure to stressors, a profound reorganization of the gland itself that prepares it to anticipate further challenge by stress, and/or to protect the integrity of the gland from deleterious effects of increased functional load. At this time, it is unclear whether acetylcholine, PACAP or a combination of the two transmitters mediates this organotypic adaptive response.

The role of gap junctions in adrenomedullary function is likely to be a generalizable phenomenon, not only to other endocrine organs (87) but also to neuronal communication: the stress stimulus selected by Guerineau for analysis of the adrenal medulla is one that is relatively slight for that organ (88) but plays a major role in regulation of norepinephrine output from post-ganglionic sympathetic nerves. It remains to be seen if principal ganglion cells of the sympathetic nervous system respond to stress as the adrenal medulla does, and if PACAP, or acetylcholine, or both are the principal regulator(s) of this important phenomenon. Of utmost importance, this work in aggregate reminds investigators of the limitations of studying endocrine phenomena in isolated cells, in which there is much insight to be gained, but in which much about how endocrine tissues actually function *in vivo* can be overlooked.

## Adrenomedullary Function During Inflammatory Responses

The inflammatory response is complex and involves virtually all organs, as cytokines are both secreted into the bloodstream and affect tissues hormonally, and because cytokine-secreting cells of the immune system are mobile and ubiquitous, migrating to various locations in response to local and systemic inflammatory challenge. The discovery of LPS and cytokine receptors on chromaffin cells was a key event in focusing on the role of the adrenal medulla in coordinating some aspects of the immune response (Figure 1). Working out the pathways for cytokine signaling and its ramifications for chromaffin cell parachymal function, and adrenomedullary participation in systemic inflammation, has been an intriguing enterprise, and worth noting within the purview of “what's new in endocrinology.”

It was noted a number of years ago that neuropeptide biosynthesis is complexly modulated by the cytokines IL-1 and TNF-alpha in bovine chromaffin cells in culture, with positive regulation of VIP biosynthesis and down-regulation of met-enkephalin, and amplification of the effects of cyclic AMP elevation on VIP and substance P production by both cytokines (89). More recently, Anouar and colleagues demonstrated the existence of TNF-alpha receptor expression in the bovine adrenal medulla *in vivo* (90, 91), and the regulation of gene transcription and peptide production of galanin and chromogranin in response to this cytokine (91). Subsequently, Bunn and colleagues postulated a role for TNF in inflammation-induced VIP biosynthesis and TH induction in rodent adrenal medulla *in vivo* (9, 92). A number of other studies converge to create a picture of cytokine regulation of adrenomedullary peptide production for a counter-regulatory role in inflammation: exposure of chromaffin cells to cytokines or administration of LPS *in vivo* (S. Bunn, personal communication) results in enhanced production of galanin, which is reported in turn to be a positive regulator of glucocorticoid production in the adrenal cortex (see Figure 2C). The inflammatory cytokines IL-1 and TNF (9) also increase the production of IL-6 mRNA in chromaffin cells, revealing an occult source of this anti-inflammatory peptide that implicates a second mechanism of down-regulation of the “cytokine storm” associated with inflammation. This anti-inflammatory mechanism is also linked to the stress-transducing function of the adrenal gland, emphasizing further the highly integrative role of the adrenal medulla as stress transducer across multiple other (metabolic, neuronal, immune) endocrine regulatory domains (Figures 1, 2).

## Summary And Future Prospects

The simple and classical view that the adrenal medulla releases catecholamines under stress in response to acetylcholine release from the splanchnic nerve provided a framework for much fruitful investigation of the detailed cellular physiology underlying the function of the chromaffin cell. This view has been enriched over the past 20 years, as summarized in Figures 1, 2. The adrenal medulla is now viewed as a more complex, and more integrative, stress transducer. Basal secretion, under the influence solely of acetylcholine, is seen as important to cardiovascular function as well. Additional first messengers besides acetylcholine, including PACAP and cytokines, bring organismic information to the chromaffin cell. The secretory products of the chromaffin cells also have important, yet-to-be-discovered paracrine, autocrine and hormonal roles. These allow integration of immune, inflammatory, sensory, and cardiovascular surveillance and regulation by the adrenal medulla. Chromaffin cells themselves not only operate as individual cellular elements that amplify signal transduction by being organized into an endocrine organ, but also modulate the performance of this cellular cohort by chromaffin cell-chromaffin cell coordination through gap junctions. Each of these themes is likely to be generalizable to other endocrine organs to some degree: PACAP at sympathetic ganglia as well as the sympathoadrenal junction; cytokine regulation of other peripheral endocrine depots such as the pancreas; gap junction function in pituitary and likely elsewhere; parcellated cAMP signaling in pancreas and brain as well as the adrenal; catestatin function throughout the cardiovascular system. Leveraging the sense that ‘everything is connected to everything else’ (93) does not mean, however, that the mammalian endocrine network is chaotic, or therapeutically opaque. Rather, apprehending directionality in endocrine regulation probably requires a better sense of what is unique about each endocrine organ, including the adrenal medulla, in addition to the predominant hormone that is released from it.

Some rather large questions remain, both about the adrenal medulla in particular, and its characteristics as an endocrine organ in general. What are the ways in which acetylcholine and PACAP collaborate in adrenomedullary function in homeostasis and stress responding? Are these two messengers specialized for “basal” or “rest and digest” vs. “stress” or “fight or flight” responding, or is their interaction more intricate than that? Does acetylcholine/PACAP co-transmission contribute importantly to transmission elsewhere in the autonomic nervous system, i.e., at both sympathetic and parasympathetic synapses? Is the adrenomedullary stress response “looped in” to both the immune-inflammatory and sensory nervous systems? Might this be a potential key to immune and sensory regulation in stress and the balance between pro- and anti-inflammatory regulation that allows glucocorticoids and cytokines to counter-balance without either being overcome in sepsis or autoimmune dysregulation? Might signaling via catestatin, BAM-22P, substance P, galanin and other secretory products of the adrenal medulla lead to regulatory sequelae that can be exploited in disease treatment? Are the signaling mechanisms newly discovered in the adrenal medulla generalizable to other endocrine organs and even the nervous system? Is metabologenomics of the chromaffin cell a paradigm that will create diagnostic/prognostic opportunities for endocrinopathies beyond pheochromocytoma (94, 95)? Finally, we would be remiss not to note again that the adrenal medulla is an organ with pronounced mammalian species differences: how much of what we have learned about its function in rodents and other mammals is directly applicable to *H. sapiens*? It is our hope that this bouquet of questions will stimulate further research leading to the discovery of new aspects of chromaffin cell function. These in turn will beget additional questions, keeping the adrenal medulla at the forefront of endocrinology, despite our recurrent conviction that we know all we need to know about this deceptively simple endocrine organ.

## Author's Note

Much of the content of this review concerns work performed in our own and colleagues' laboratories and its precedents that are only now emerging in the literature. We apologize in advance for any myopia about progress in chromaffin cell endocrinology emerging from other laboratories that this review may have overlooked.

## Author Contributions

All authors listed have made a substantial, direct and intellectual contribution to the work, and approved it for publication.

### Conflict of Interest Statement

The authors declare that the research was conducted in the absence of any commercial or financial relationships that could be construed as a potential conflict of interest.

## Footnotes

^1^As often as possible, in the interests of brevity, review articles in which the historical documentation of discovery can be perused, will be cited in lieu of the primary literature.
